# Distribution of the N_2_ ‐fixing cyanobacterium *Candidatus* Atelocyanobacterium thalassa in the Mexican Pacific upwelling system under two contrasting El Niño Southern Oscillation conditions

**DOI:** 10.1111/1758-2229.13237

**Published:** 2024-02-13

**Authors:** Cinthya Vieyra‐Mexicano, Valeria Souza, Silvia Pajares

**Affiliations:** ^1^ Unidad Académica de Ecología y Biodiversidad Acuática, Institute of Marine Sciences and Limnology National Autonomous University of Mexico Mexico City Mexico; ^2^ Posgrado en Ciencias del Mar y Limnología National Autonomous University of Mexico Mexico City Mexico; ^3^ Departamento de Ecología Evolutiva, Institute of Ecology National Autonomous University of Mexico Mexico City Mexico

## Abstract

The unicellular cyanobacterium *Candidatus* Atelocyanobacterium thalassa (UCYN‐A) is a key diazotroph in the global ocean owing to its high N_2_ fixation rates and wide distribution in marine environments. Nevertheless, little is known about UCYN‐A in oxygen‐deficient zones (ODZs), which may be optimal environments for marine diazotrophy. Therefore, the distribution and diversity of UCYN‐A were studied in two consecutive years under contrasting phases (La Niña vs. El Niño) of El Niño Southern Oscillation (ENSO) along a transect in the ODZ of the Mexican Pacific upwelling system. Of the three UCYN‐A sublineages found, UCYN‐A1 and UCYN‐A3 were barely detected, whereas UCYN‐A2 was dominant in all the stations and showed a wide distribution in both ENSO phases. The presence of UCYN‐A was associated with well‐oxygenated waters, but it was also found for the first time under suboxic conditions (<20 μM) at the bottom of a shallow coastal station, within the oxygen‐poor and nutrient‐rich Subsurface Subtropical water mass. This study contributes to the understanding of UCYN‐A distribution under different oceanographic conditions associated with ENSO phases in upwelling systems, especially because of the current climate change and increasing deoxygenation in many areas of the world's oceans.

## Graphical abstract

This article expands our understanding of the distribution patterns of the UCYN‐A clade in upwelling systems. Here, the influence of oceanographic variability in shaping the distribution of UCYN‐A was investigated along a coast‐ocean transect in the Mexican Pacific upwelling system under two contrasting ENSO conditions (La Niña vs. El Niño). Three UCYN‐A sublineages were observed in the study region: UCYN‐A1 and UCYN‐A3 were barely detected, whereas UCYN‐A2 was dominant with a wide distribution in both ENSO phases. Furthermore, the punctual presence of UCYN‐A in nutrient‐rich suboxic waters in a shallow coastal station is reported for the first time.

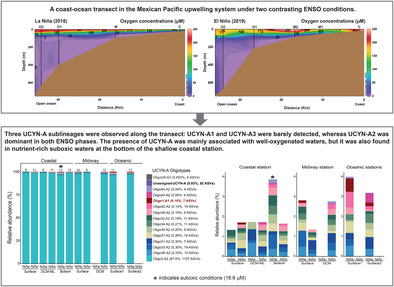

## INTRODUCTION

Biological nitrogen (N) fixation provides the largest external input of N to the ocean, fuelling phytoplanktonic photosynthesis and regulating the subsequent export of organic matter into the deep waters through the biological carbon pump (Zehr & Capone, 2020). It is an energetically expensive process performed by a particular group of prokaryotes called diazotrophs that convert N_2_ into biologically available ammonia through the nitrogenase complex, which is irreversibly inactivated by molecular oxygen (Karl et al., 2002). Molecular techniques based on the amplification of *nifH*, the marker gene for diazotrophy, have revealed that certain cyanobacteria and non‐cyanobacterial diazotrophs fix N_2_ in distinct marine systems and are more abundant than previously thought (e.g., Bombar et al., 2016; Moisander et al., 2010; Zehr, 2011; Zehr et al., 1998). For example, the unicellular cyanobacterial group *Candidatus* Atelocyanobacterium thalassa (UCYN‐A) is a key component of the global marine diazotrophic community and can achieve high rates of biological N fixation in the ocean surface (Gradoville et al., 2020; Martínez‐Pérez et al., 2016; Tang et al., 2020; Turk‐Kubo et al., 2021).

UCYN‐A lives symbiotically with a single‐celled haptophyte alga related to *Braarudosphaera bigelowii* (Hagino et al., 2013; Thompson et al., 2012). The basis of this symbiosis is the transfer of fixed carbon from the host in exchange for fixed N (Thompson et al., 2012) since UCYN‐A lacks oxygenic photosynthesis, carbon fixation and many other essential metabolic pathways (Tripp et al., 2010; Zehr et al., 2008). Eight UCYN‐A sublineages (UCYN‐A1 to ‐A8) have been defined based on the phylogenetic diversity of *nifH* gene sequences (Farnelid et al., 2016; Henke et al., 2018; Thompson et al., 2014; Turk‐Kubo et al., 2017), of which UCYN‐A1 to ‐A4 are known to occupy different ecological niches (Farnelid et al., 2016; Turk‐Kubo et al., 2017).

UCYN‐A is of widespread importance in the ocean as it has been recorded in diverse marine environments (e.g., Krupke et al., 2014; Li et al., 2021; Shao et al., 2023; Shiozaki et al., 2017; Tang et al., 2020; Turk‐Kubo et al., 2017), including regions not typically assumed to be important for biological N fixation, such as N‐enriched waters, especially polar seas (Harding et al., 2018; Shiozaki et al., 2018), coastal waters (Cabello et al., 2020; Mulholland et al., 2012) and upwelling regions (Agawin et al., 2014; Moreira‐Coello et al., 2019; Selden et al., 2022; Turk‐Kubo et al., 2021). However, UCYN‐A has not been detected in oxygen‐deficient zones (ODZs) under suboxic conditions (<20 μM), which may represent a favourable niche for N_2_ fixation, since in these environments diazotrophs do not need to expend additional energy for oxygen removal from the surrounding water to protect nitrogenase (Fay, 1992; Robson & Postgate, 1980). Furthermore, the high nitrate (NO_3_^−^) concentration often present in ODZs may not inhibit the growth of the UCYN‐A/haptophyte symbiosis, which can fix N_2_ in NO_3_^−^‐rich waters (Mills et al., 2020), an ability also observed in *Crocosphaera watsonii* (UCYN‐B) (Dekaezemacker & Bonnet, 2011; Großkopf & LaRoche, 2012). To date, UCYN‐B and non‐cyanobacterial diazotrophs have been found in oxygen‐deficient waters (Bonnet et al., 2013; Fernandez et al., 2011; Jayakumar et al., 2017; Jayakumar & Ward, 2020; Loescher et al., 2014; Löscher et al., 2020), whereas UCYN‐A has been detected in the Eastern Tropical North Pacific (ETNP), the Eastern Tropical South Pacific (ETSP) and the Northern Benguela Upwelling system, but only within their oxygenated waters (Reeder et al., 2022; Turk‐Kubo et al., 2014, 2021; White et al., 2013). Little is known about the ecology of UCYN‐A in ODZs, therefore, providing information on its distribution in these oceanic regions is of utmost importance for a better understanding of the players involved in N fixation in ODZs.

The paucity of oxygen in ODZs is generated by physicochemical and biological processes such as thermal stratification, poor circulation and the upwelling of nutrient‐rich waters to the surface that drive biological productivity and oxygen consumption (Paulmier & Ruiz‐Pino, 2009). The main ODZs are found in the Arabian Sea and along the eastern boundaries of the Pacific Ocean in the ETNP and the ETSP (Paulmier & Ruiz‐Pino, 2009). Despite constituting only ∼7% of the global oceanic volume, ODZs are responsible for 30%–50% of the marine N loss, having a significant impact on the oceanic N balance (DeVries et al., 2013). In addition, these regions are expanding because of global warming and anthropogenic activities, thereby increasing ocean deoxygenation and affecting marine N biogeochemistry (Hutchins & Capone, 2022; Levin, 2018).

The ODZs are further exacerbated in upwelling areas, especially in the ETNP, which represents one of the most extensive and intense ODZs in the global ocean (Paulmier & Ruiz‐Pino, 2009). This region is subjected to the inter‐annual climactic variability of El Niño Southern Oscillation (ENSO), which modulates the strength of the upwelling and the ODZ upper boundary (Yang et al., 2017), regulating prokaryotic assemblages and N‐cycling genes (Pajares et al., 2023). Biological N fixation rates have been observed with a patchy distribution in oxygen‐deficient waters of the ETNP (Selden et al., 2019). However, the distribution and dynamics of the diazotrophic community in this region are less well‐known than in other ODZs, particularly in the coastal waters off Mexico (Jayakumar et al., 2017; White et al., 2013).

In this research, the spatiotemporal distribution and diversity of UCYN‐A sublineages were studied by comparison with existing UCYN‐A oligotypes (alternative taxonomic units defined by nucleotide positions with high variability) along a transect in the ODZ of the Mexican Pacific during the upwelling season under two consecutive years with different ENSO conditions, La Niña (cold phase) and El Niño (warm phase). We hypothesized that the distribution of UCYN‐A changes according to different oceanographic conditions associated with two consecutive ENSO phases. Likewise, we expected spatial differences in the composition and structure of the UCYN‐A community along the transect and over depths.

## EXPERIMENTAL PROCEDURES

### Study location, sample collection and chemical analyses

**FIGURE 1:**
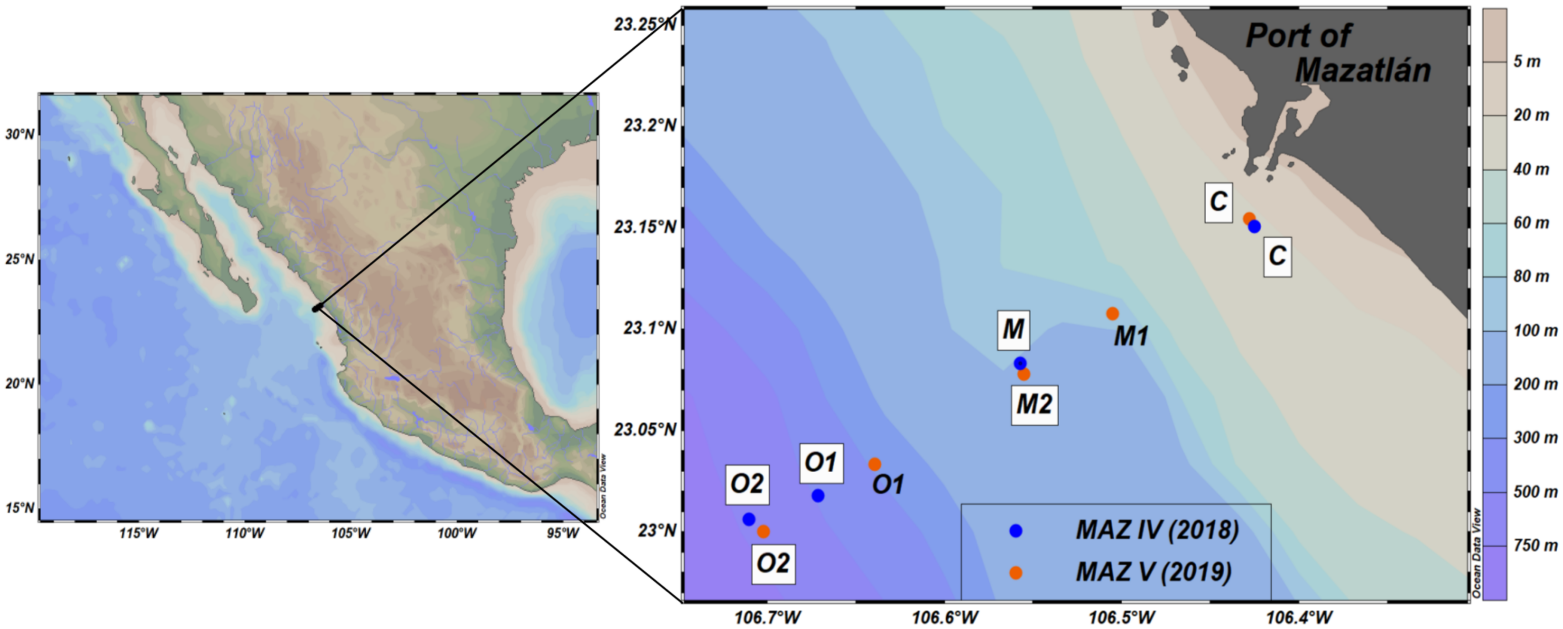
Sampling stations along the transect in the ETNP off Mexico. Symbol colours denote the two cruises. White boxes: stations for molecular analysis of UCYN‐A. Stations: C, coastal; M, midway (midway 1 and 2 in MAZ V); O, oceanic 1 and 2 in both cruises. Depths: from 32 m in the coastal station to an average of 690 m in the most oceanic station.

The study area was in the Mexican Pacific within the ODZ of the ETNP (Figure 1). Hydrographic data and seawater samples were collected along a transect extending from nearshore to offshore waters in front of the Port of Mazatlán during the oceanographic cruises MAZ IV (April 2018, La Niña) and MAZ V (April 2019, El Niño) on board R/V ‘El Puma’ of the National Autonomous University of Mexico (UNAM). The transect consisted of four stations during MAZ IV and five stations during MAZ V for oceanographic and nutrient analyses, but only labelled stations on the map were considered for molecular analysis: four stations during MAZ IV and three stations during MAZ V (Figure 1).

Profiles of temperature, dissolved oxygen, salinity and chlorophyll‐*a* (fluorescence) were obtained with an SBE‐19plus CTD probe equipped with a fluorometer sensor (WET labs ECO‐AFL/FL). Seawater samples for molecular analysis were collected in 10‐L Niskin bottles deployed on a CTD rosette based on the oxygen and chlorophyll‐*a* profiles: surface (5 m depth); deep chlorophyll‐*a* maximum (DCM, at 10–45 m depth, except at the coastal station of MAZ V where the water column was mixed and a sample was collected from the mixed layer, ML); base of the oxycline (60–125 m depth); ODZ core (250–400 m depth in the oceanic stations); and bottom (between 23 m depth in the coastal station and 670 m depth in the most oceanic station). In addition, samples for nutrient analysis were collected from specific depths (Table S1).

Duplicated seawater samples for nutrient analyses were processed as described elsewhere (Pajares et al., 2023). Dissolved nutrient concentrations (ammonium (NH_4_^+^), nitrite (NO_2_^−^), NO_3_^−^ and soluble reactive phosphorus (PO_4_^−3^)) were determined with a San‐Plus segmented‐flow autoanalyser (Skalar Analytical B.V.) and the methods adapted for seawater analysis by Hansen and Koroleff (1999). The detection limits for these analyses were 0.1 μM for NH_4_^+^, 0.02 μM for NO_2_^−^, 0.1 μM for NO_3_^−^ and 0.04 μM for PO_4_^−3^.

Triplicate 1.3 L seawater samples for molecular analysis were filtered on board through polycarbonate membranes (0.22 μm pore, Merck Millipore). Filters were frozen at −80°C until DNA analysis.

### DNA extraction and qPCR assay

DNA was extracted from the filters with the DNeasy PowerWater Kit (Qiagen), quantified on a Qubit 4.0 Fluorometer (Thermo Fisher Scientific) and diluted to 5 ng/μL for the qPCR assay.

The abundance of the UCYN‐A *nifH* gene was quantified by qPCR with the univ_UCYN‐A_F_CS1/univ_UCYN‐A_R_CS2 primers (Turk‐Kubo et al., 2017). Reactions were performed in 20 μL of reaction mixture containing ×1 SYBR Green PCR Master Mix (Applied Biosystems), 0.5 μM primers, 0.2 mg/mL BSA (Thermo Fisher Scientific) and 0.25 ng/μL DNA. Thermal cycling was performed on a StepOnePlus Real‐Time PCR System (Applied Biosystems) as follows: 5 min at 95°C, 40 cycles of 30 s at 95°C, 30 s at 55°C and 30 s at 72°C, followed by a final elongation of 7 min at 72°C. Reactions were performed in triplicate, and the standard curves consisted of serial 10‐fold dilutions of a linearized plasmid containing the targeted *nifH* insert. The specificity of the amplified products was confirmed by melt‐curve examination and agarose gel electrophoresis. Efficiency and correlation coefficients were 97.4% and 0.99, respectively.

### *UCYN‐A* nifH *gene sequencing*

A nested PCR was performed to amplify UCYN‐A *nifH* gene with the universal *nifH* primers *nifH3*/*nifH4* (Zani et al., 2000) for the first round and the UCYN‐A *nifH* specific primers univ_UCYN‐A_F_CS1/univ_UCYN‐A_R_CS2 (Turk‐Kubo et al., 2017) for the second round. PCR reactions contained ×1 MyTaq buffer (1 mM dNTPs, 3 mM MgCl_2_), 0.5 μM primers, 0.2 mg/mL BSA and 0.025 U/μL MyTaq polymerase (Bioline). Thermal cycling for the first PCR was performed as follows: 5 min at 95°C, 35 cycles of 1 min at 95°C, 1 min at 57°C and 1 min at 72°C, followed by a final elongation of 7 min at 72°C. The second PCR was performed with the same thermal cycling conditions as the qPCR but with 35 cycles. PCR reactions were performed in duplicate on all DNA samples.

DNA amplicons were purified with the QIAquick Gel/PCR band purification kit (Qiagen) and quantified on a Qubit 4.0 Fluorometer. Purified PCR products with a minimum DNA concentration of 10 ng/μL were sequenced on a paired‐end (2 × 300 bp) Illumina MiSeq platform at the National Laboratory of Genomics for Biodiversity (Center for Research and Advanced Studies, Mexico).

### Bioinformatic analyses and taxonomic assignment

Single‐end reads were processed using the Quantitative Insight Into Microbial Ecology 2 pipeline (QIIME 2 v. 2021.2, Bolyen et al., 2019). Reads were truncated based on sequencing quality, denoised and dereplicated, and chimeras were filtered with the DADA2 plugin (Callahan et al., 2016). In total, 1383 amplicon sequence variants (ASVs) were generated from 3,868,894 sequences that passed all quality filtering steps with an average sequence length of 255 bp. Subsequently, ASVs were sorted with a *nifH* gene reference database (*nifH*_dada2_all_v1.fasta; Moynihan, 2020), based on the ARB database from Zehr's Laboratory (version June 2017; https://www.jzehrlab.com/nifH), using the ‘assignTaxonomy’ tool of DADA2 package v. 1.20.0 (Callahan et al., 2021) in RStudio v. 1.4.1103 (RStudio Team, 2021). In total, 498 sequences (43 ASVs) not assigned to any reference *nifH* sequence were removed from the dataset. Subsequently, the UCYN‐A sublineages were assigned with the ‘assignTaxonomy’ tool of DADA2 by comparison with reference sequences of UCYN‐A oligotypes belonging to the North and South Atlantic, North and South Pacific and Denmark Strait data set (Turk‐Kubo et al., 2017). In both taxonomic assignments, the ‘tryRC’ parameter was used to assess whether the reverse‐complement orientation of each sequence matched the reference sequences better than the forward orientation. Finally, the ASVs table was rarefied to 234,869 sequences per sample (the minimum sequencing depth) in QIIME 2, obtaining 1340 ASVs.

### Phylogenetic and diversity analyses

The ClustalW algorithm in BioEdit v. 7.0.5.3 (Hall, 1999) aligned the most abundant representative ASVs from each oligotype along with published reference sequences from the NCBI database belonging to UCYN‐A oligotypes and three reference sequences from UCYN‐B and UCYN‐C as out‐groups (Cornejo‐Castillo et al., 2019; Thompson et al., 2014; Turk‐Kubo et al., 2017). A Maximum Likelihood tree was constructed in MEGA‐X (Kumar et al., 2018) based on the Tamura‐Nei model and 1000 bootstrap replicates. The phylogenetic tree was edited and visualized with the interactive Tree of Life (iTOL) online program (Letunic & Bork, 2016).

An upset diagram with the UpSetR package v. 1.4.0 (Conway et al., 2017) and a Venn diagram with the VennDiagram package v. 1.7.3 (Chen, 2022) in RStudio determined the number of unique and shared ASVs among ENSO conditions, depths and sampling stations. Estimation of alpha diversity indices (Chao1, Shannon, Simpson 1‐D and Faith's PD) used the standardized abundance table of ASVs with the vegan package v. 2.5‐7 (Oksanen et al., 2020) in RStudio. Finally, a redundancy analysis (RDA) with the vegan package estimated the degree of linear association between the UCYN‐A community and the environmental variables. The abundance table of ASVs was transformed by the Hellinger method and the environmental variables were logarithmically transformed before use in RDA (Legendre & Gallagher, 2001). To minimize the collinearity of explanatory variables in the RDA model, a subset of environmental variables was chosen according to their variance inflation factor (VIF < 10). The contribution of each selected variable was evaluated using a permutation test. The RDA plot was generated with the ggplot2 package v. 3.3.5 (Wickham et al., 2021).

## RESULTS AND DISCUSSION

### Oceanographic conditions of the ODZ in the Mexican Pacific

**FIGURE 2:**
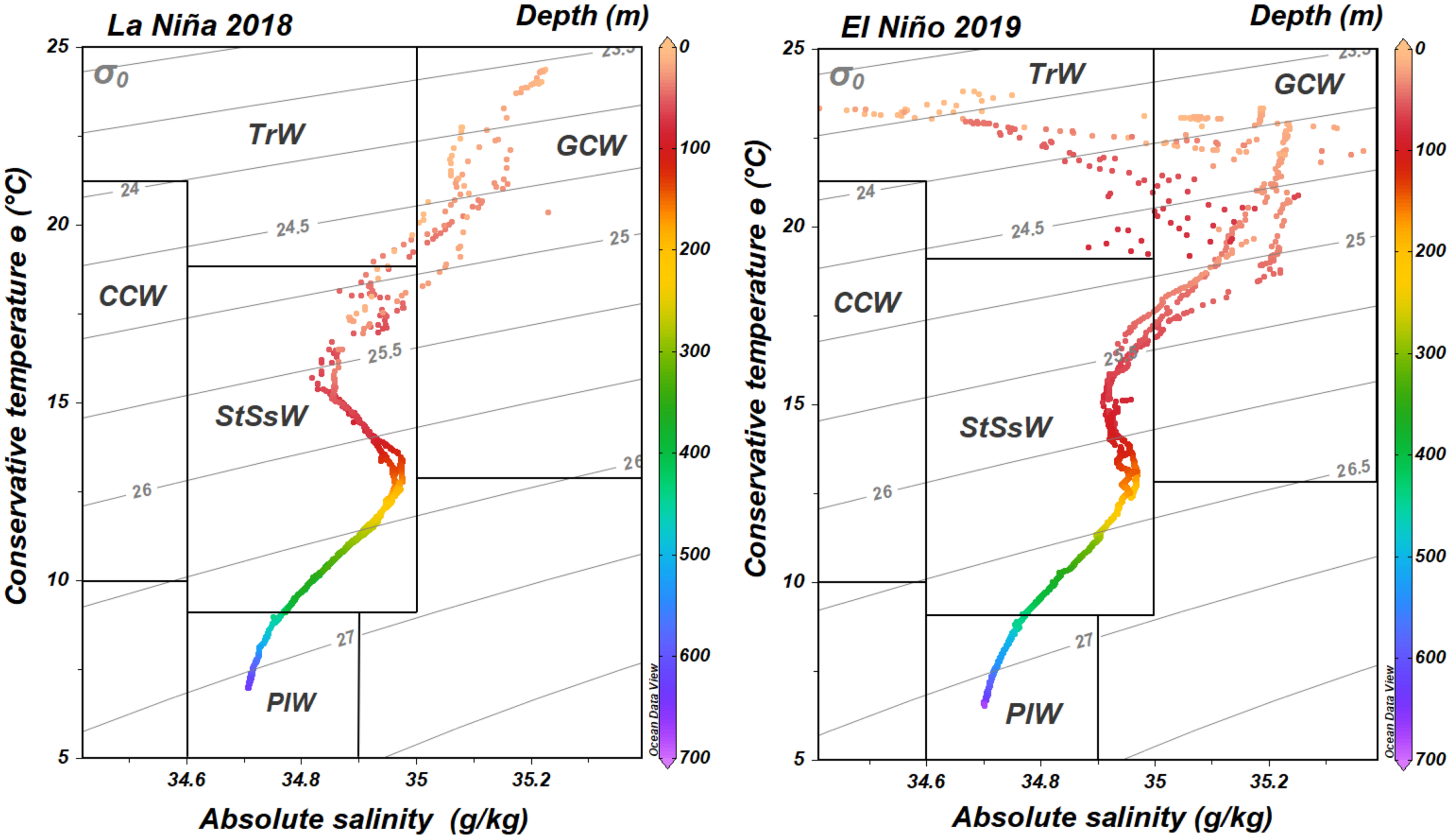
Temperature‐salinity diagrams for all sampling stations during both ENSO phases. CCW, California Current water; GCW, Gulf of California water; PIW, Pacific Intermediate water; StSsW, Subsurface Subtropical water; TrW, Transitional water.

According to the criteria defined by Lavín and Marinone (2003) and Portela et al. (2016), four water masses were recognized during both cruises in the study area (Figure 2): (1) Gulf of California water (GCW, oxygenated surface waters with temperature >12°C and absolute salinity >35 g/kg), which had a higher proportion during El Niño (40–75 m depth) than during La Niña (0–35 m depth); (2) Subsurface Subtropical water (StSsW, intermediate waters with low oxygen, temperature of 9°C–18°C and absolute salinity of 34.6–35 g/kg), which extended to ~420–440 m depth in the oceanic stations during both ENSO phases and reached the shallow coastal station (from 17 m depth to the seafloor) during La Niña; (3) Transitional water (TrW, derived from the mixture of GCW and StSsW); (4) Pacific Intermediate water (PIW, anoxic waters with temperature of 4°C–9°C and absolute salinity of 34.6–34.9 g/kg) below the StSsW mass down to the seafloor in the most oceanic station.

Sea surface temperature varied according to the presence of La Niña in 2018 and El Niño in 2019 (https://psl.noaa.gov/enso/mei/). A stronger upwelling during La Niña brought the StSsW mass, with high‐nutrient and oxygen‐deficient waters, towards the coast. In contrast, the westward movement of the warm GCW mass during El Niño led to a weaker upwelling, promoting a displacement of the thermocline to deeper waters and increasing oxygenation and salinity (Figure S1). Therefore, oxygen concentration at the sea surface was high (>200 μM), but it decreased towards the coast during La Niña (191 μM) owing to the influx of low‐oxygen waters. In the shallow coastal station, the onset of the ODZ (O_2_ <20 μM) was at 17 m depth during La Niña, whereas oxygen was saturated throughout the water column during El Niño. In most oceanic stations, the onset of the ODZ was at a shallower depth (∼68 m) during La Niña than during El Niño (∼80 m depth).

Chlorophyll‐*a* had higher values during La Niña (6.2 mg/m^3^ at 10 m depth nearshore; 2 mg/m^3^ at 30 m depth offshore), owing to the intense upwelling than during El Niño (1.6 mg/m^3^ at 23 m depth nearshore; 1.5 mg/m^3^ at 38 m depth offshore). In general, nutrient distributions (NH_4_^+^, NO_2_^−^, NO_3_^−^, PO_4_^3−^) throughout the water column showed typical patterns of an ODZ and were like those previously reported in the Mexican Pacific (Jayakumar et al., 2017; Pajares et al., 2019; Selden et al., 2019). Nutrient concentrations were higher during La Niña than during El Niño because of the upwelling (Figure S2; Pajares et al., 2023).

### Composition, diversity and spatiotemporal distribution of UCYN‐A

Using a UCYN‐A‐specific approach, the UCYN‐A *nifH* gene was amplified, quantified and sequenced throughout the water column (surface, DCM/ML and bottom samples) in the shallow coastal station (C) during both ENSO phases (Table S1). These coastal samples were under the dominance of the oxygenated TrW (La Niña) and GCW (El Niño) masses, except the bottom sample during La Niña that was within the oxygen‐poor StSsW mass. The gene was also amplified and quantified from all surface (within the GCW mass) and DCM samples (within the TrW mass, except the most oceanic sample during El Niño that was within the GCW mass) of the midway (M and M2) and oceanic (La Niña: O1 and O2; El Niño: O2) stations in both ENSO phases. However, it could only be sequenced from the surface samples during La Niña (stations M, O1, O2) and from the surface and DCM samples of the midway station (M2) during El Niño because the concentration of the PCR products was insufficient in the other samples. Finally, as expected, no sample from the aphotic zone was amplified.

**FIGURE 3:**
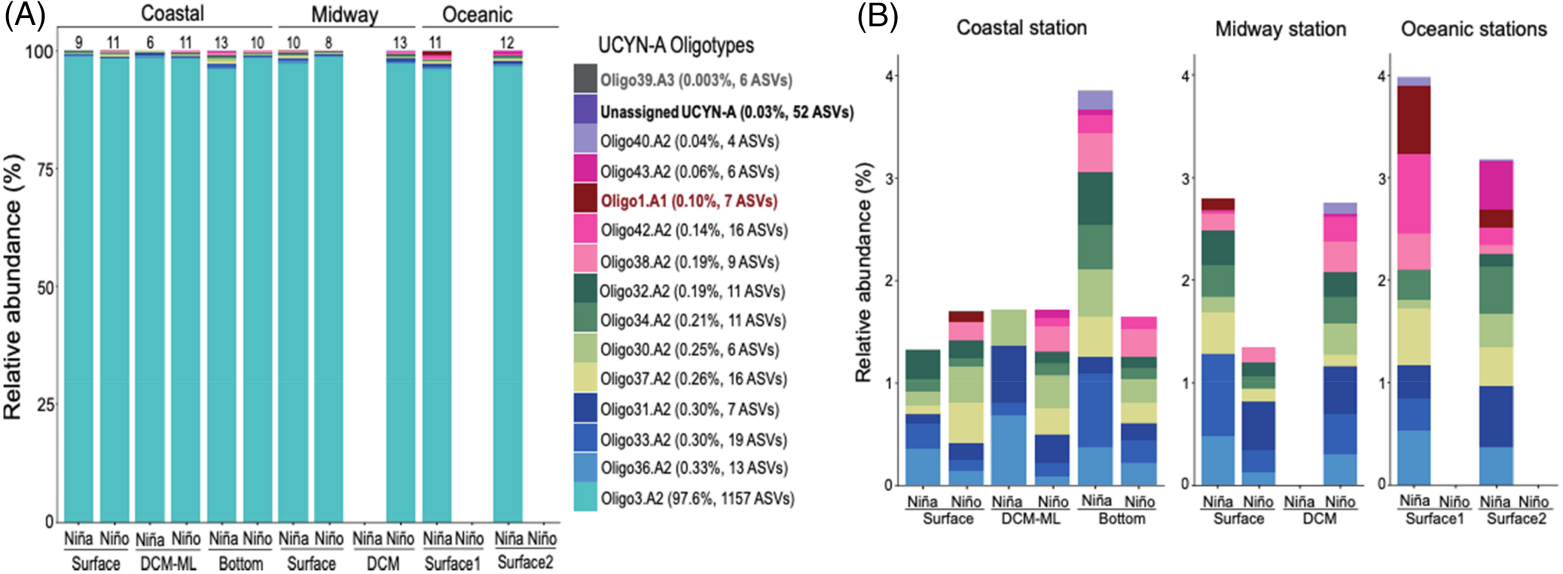
(A) Relative abundance of UCYN‐A oligotypes in the *nifH* sequences from the sampling stations along the transect during the two consecutive ENSO phases. Above each bar, the number of oligotypes identified in each sample. (B) Relative abundance of the less abundant oligotypes (excluding oligo3 and unassigned UCYN‐A sequences) in each section of the transect during the two ENSO phases. Above the bars, sampling sections; below the bars, ENSO phases and sampling depths. Missing bars, samples not sequenced. DCM, deep chlorophyll‐*a* maximum; ML, mixed layer.

Of the total 1340 ASVs assigned to *nifH* sequences, 52 ASVs belonged to UCYN‐A sequences unassigned to any sublineage (0.03% of the total reads) and 1288 ASVs were assigned to three UCYN‐A sublineages (99.97% of the total reads) (Figure 3; Table S2): UCYN‐A2 was by far the most abundant sublineage along the study transect (99.87% of the total UCYN‐A sequences), whereas UCYN‐A1 and UCYN‐A3 had a very low relative abundance (0.1% and 0.003% of the total UCYN‐A sequences, respectively). The remarkably high relative abundance of UCYN‐A2 found here is in good agreement with the relative abundances of UCYN‐A2 reported in other upwelling regions (e.g., Cabello et al., 2020; Moreira‐Coello et al., 2019). However, it cannot be ruled out that its high abundance might be due, in part, to a bias of the primers used in this study (Turk‐Kubo et al., 2017), since the specificity of the primers may influence the portion of the community detected. However, studies in different marine environments have also found a high abundance of this sublineage using other primer sets (e.g., Cabello et al., 2020; Li et al., 2021; Moreira‐Coello et al., 2019).

The UCYN‐A2 sublineage was found in high relative abundance in all sequenced samples along the transect (Figure 3), in which the concentrations of dissolved inorganic N (DIN) ranged from 0.35 to 14.7 μM during La Niña and from 0.62 to 1.6 μM during El Niño (Figure S2). The abundance of UCYN‐A2 and its haptophyte host has been shown to increase in response to upwelling with high NO_3_^−^ concentrations in surface waters of the coastal Arctic Ocean, suggesting that this symbiosis thrives well following upwelling‐driven nutrient pulses (Selden et al., 2022), as appears to be the case in this study area and other upwelling systems globally (e.g., Moreira‐Coello et al., 2019; Turk‐Kubo et al., 2021). However, no significant relationship has been observed between upwelling conditions and UCYN‐A2 abundance off the Southern California coast (Fletcher‐Hoppe et al., 2023). In addition, other studies have reported a wide distribution of UCYN‐A2, including oligotrophic oceanic zones and nutrient‐rich coastal waters (Li et al., 2021; Shiozaki et al., 2017; Turk‐Kubo et al., 2017), in which it was not excluded under high DIN availability (Mills et al., 2020).

The presence of UCYN‐A2 off Mazatlán at temperatures ranging from 17.3°C to 24.4°C agrees with records from other regions (Cabello et al., 2020; Thompson et al., 2014; Turk‐Kubo et al., 2021). However, it has also been recovered from the cold waters of the Arctic Ocean (Harding et al., 2018; Selden et al., 2022; Shiozaki et al., 2018). In the Mazatlán transect, UCYN‐A2 was detected mostly in oxygenated waters (>118 μM), but also in low‐oxygen waters of the coastal station during La Niña (60 μM at DCM—10 m depth and 16.6 μM at the bottom—23 m depth). However, its punctual detection in oxygen‐deficient waters does not mean that this sublineage is active, since it may be trapped on sinking particles, as previously observed in the North Pacific Subtropical Gyre (Farnelid et al., 2019). Thus, although the mechanisms driving UCYN‐A2 distribution are complex and not yet fully understood, this sublineage seems to tolerate a wide range of conditions in this region of the ETNP.

The UCYN‐A1 sublineage was detected at very low relative abundance at the surface of the midway (M) and oceanic stations (O1 and O2) during La Niña and at the surface of the coastal (C) station during El Niño, within the oligotrophic well‐oxygenated GCW mass (Figure 3). UCYN‐A1 might have also been present at the surface of the oceanic station during El Niño, but the PCR product from this sample was insufficient to be sequenced. A recent study at the San Pedro time series off Southern California found that UCYN‐A1 was negatively influenced by seasonal upwelling and increased its abundance after weak upwelling conditions triggered by El Niño (Fletcher‐Hoppe et al., 2023), which may explain why this sublineage was barely detected in this study. Furthermore, UCYN‐A1 has been found typically in open ocean waters, where the oligotrophic conditions seem to favour its presence (Gradoville et al., 2020; Shiozaki et al., 2017; Turk‐Kubo et al., 2017, 2021). However, previous findings indicate that UCYN‐A1 can co‐occur with UCYN‐A2 in coastal waters (Cabello et al., 2020; Henke et al., 2018; Moreira‐Coello et al., 2019; Stenegren et al., 2018; Thompson et al., 2014), but such co‐occurrence could strongly vary both interannually and on weekly timescales (Robicheau et al., 2023). Thus, these sublineages might share a similar niche in some circumstances, although it remains unclear whether these sites represent overlapping niches for UCYN‐A1 and UCYN‐A2 or whether the presence of UCYN‐A1 in coastal waters (as in this study during El Niño) is the result of the intrusion of oligotrophic waters (Cabello et al., 2020).

UCYN‐A3 was absent in this dataset. This sublineage was barely detected at the surface and the bottom of the coastal (C) station during La Niña, as well as at the surface of the coastal (C) station and the DCM of the midway (M) station during El Niño (Figure 3). So far, the presence of the UCYN‐A3 sublineage has been sporadically reported in different regions, such as in an inverse estuary in southern Australia (Messer et al., 2015), in open ocean waters (Turk‐Kubo et al., 2017) and offshore of the New Caledonian coral lagoon (Henke et al., 2018) and the Southern California Current System (Turk‐Kubo et al., 2021), where it often co‐occurs with UCYN‐A1.

There were temporal and spatial differences in the abundance of the UCYN‐A community off Mazatlán. For example, the highest copy number of the UCYN‐A *nifH* gene was always found at the surface during both ENSO phases (La Niña: 141–306 copies/mL, El Niño: 114–218 copies/mL; Figure S3, Table S1). These values of absolute abundance obtained by qPCR are consistent with previous reports in surface waters from different locations in the Pacific using specific primer pairs for *nifH* of UCYN‐A1 and UCYN‐A2/A3 (e.g., Cornejo‐Castillo et al., 2019; Gradoville et al., 2020; Turk‐Kubo et al., 2021), suggesting that UCYN‐A might be an important contributor to N_2_ fixation in the surface waters of this region. In addition, during both ENSO phases the copy number of UCYN‐A *nifH* was lower toward the oceanic stations, where the nutrient concentrations were also lower than nearshore. Furthermore, UCYN‐A *nifH* was slightly more abundant in all samples during La Niña (from 80 to 306 copies/mL), when the upwelling was stronger (and the concentrations of chlorophyll‐*a* and nutrients were higher), than during El Niño (from 24 to 218 copies/mL).

**FIGURE 4:**
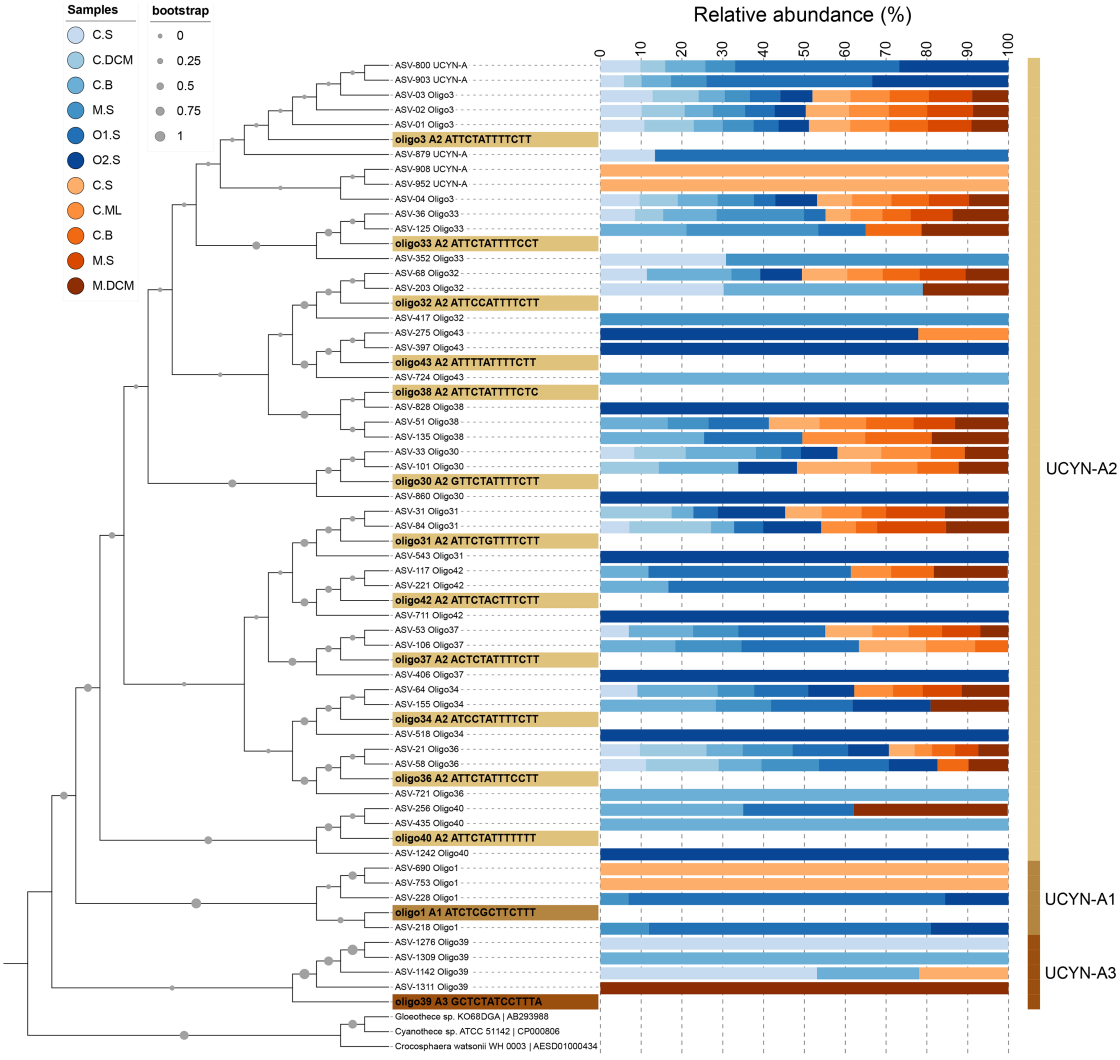
Phylogenetic tree of the 50 representative ASVs belonging to the 14 UCYN‐A oligotypes detected in this study. Reference sequences of UCYN‐A oligotypes and from closely related unicellular cyanobacteria (UCYN‐B and UCYN‐C) were also used. The grey circle at the nodes, bootstrap value. Vertical bars and highlighting labels: gold, UCYN‐A2; light brown, UCYN‐A1; brown, UCYN‐A3. Bar graph of the relative abundance of each ASV: blue gradient, samples from La Niña; orange gradient, samples from El Niño. Sample labels in the colour legend: Stations: C, coastal; M, midway; O, oceanic. Depth: S, surface; DCM, deep chlorophyll‐*a* maximum; ML, mixed layer; B, bottom.

The UCYN‐A2 sequences obtained in the samples off Mazatlán matched 12 oligotypes recognized for this sublineage by Turk‐Kubo et al. (2017), whereas the few sequences of UCYN‐A1 and UCYN‐A3 corresponded only to oligotypes 1 and 39, respectively (Figures 3 and 4). Studies in other marine systems have reported most of the UCYN‐A2 oligotypes found here, but also many more UCYN‐A1 and UCYN‐A3 oligotypes than here (Turk‐Kubo et al., 2017, 2021). For instance, in the upwelling region off northwestern Iberia, although UCYN‐A2 was by far the most abundant sublineage, formed by 19 oligotypes (with oligo3 dominant), UCYN‐A1 was formed by 21 oligotypes (with oligo1 dominant), whereas UCYN‐A3 was absent (Moreira‐Coello et al., 2019). Likewise, 60 oligotypes were found in the coral lagoon of New Caledonia, of which several belonged to UCYN‐A1 and UCYN‐A3 (Henke et al., 2018).

Although oligotype 3, belonging to UCYN‐A2, was dominant in the UCYN‐A community in this study, there were changes in the distribution of other less abundant oligotypes found here (Figure 3). For example, during La Niña a high number of oligotypes were found at the surface of the most oceanic station (1 UCYN‐A1 oligotype and 11 UCYN‐A2 oligotypes), fostered by the oxygenated and nutrient‐poor conditions of the GCW mass. A high number of oligotypes were also found in the nutrient‐rich suboxic bottom of the shallow coastal station (12 UCYN‐A2 oligotypes and 1 UCYN‐A3 oligotype, within the StSsW mass), a portion of which could come from sediment resuspension events fostered by a stronger upwelling during La Niña, which could ‘re‐seeded’ UCYN‐A populations into the water column, as previously suggested (Cabello et al., 2020). In contrast, the lowest number was found at the DCM of the same station during this phase (6 UCYN‐A2 oligotypes, within the TrW mass).

Within the UCYN‐A2 sublineage, oligotypes 3, 34 and 36 occurred in all sampling stations, but with different relative abundances (Figure 3). Oligo3 dominated by far the UCYN‐A community (97.6% of the sequences, belonging to 1157 ASVs), whereas oligotype 34 (11 ASVs) and oligotype 36 (13 ASVs) accounted for only 0.21% and 0.33% of the UCYN‐A sequences, respectively. Furthermore, the most abundant ASVs corresponding to oligo3 had a similar relative abundance distribution in all stations (e.g., ASVs 1, 2, 3 and 4 represented in the phylogenetic tree; Figure 4). Therefore, oligo3 does not appear to have defined biogeography within the study region since it was found in great abundance along the transect during both ENSO conditions. This is consistent with reports of the dominance of oligo3 among UCYN‐A2 sequences in coastal‐influenced waters such as in Monterey Bay (Cabello et al., 2020), the New Caledonian coral lagoon (Henke et al., 2018), the northwestern Iberian upwelling system (Moreira‐Coello et al., 2019), the Danish Strait and the California Current System (Turk‐Kubo et al., 2017). Further research is needed to determine whether these coastal regions represent potential niches for this UCYN‐A2‐affiliated oligotype.

UCYN‐A2 oligotypes 30, 31, 32, 33, 37, 38, 40, 42 and 43 were found here in a very low relative abundance (≤0.3% of the sequences and with 4–19 ASVs each) (Figure 3). All these oligotypes have also been found in low abundance in a wide range of oceanic regions (Turk‐Kubo et al., 2017), but little is known about their distribution in specific marine environments. For instance, these oligotypes have been found in the northwestern Iberian upwelling system (Moreira‐Coello et al., 2019), whereas oligotypes 30, 40 and 43 have also been reported in the oligotrophic waters of the New Caledonia coral lagoon (Henke et al., 2018), as well as oligotypes 37 and 40 in the Southern California Current System (Turk‐Kubo et al., 2021).

Among the 50 representative ASVs of the 14 UCYN‐A oligotypes detected in this study (Figure 4), only five were present in all the samples (ASVs 1–4 related to oligo3, and ASV 21 related to oligo36). On the other hand, during La Niña, 14 unique representative ASVs were found in the samples. Conversely, during El Niño unique representative ASVs were only found at the surface of the coastal station (4 ASVs) and the DCM of the midway station (1 ASV). In addition, the 19 representative ASVs found at the oxygenated bottom of the coastal station during El Niño were also distributed in other samples from both ENSO phases. In contrast, there were 30 representative ASVs at the suboxic bottom of this coastal station during La Niña, in the domain of the StSsW mass, of which four (ASV 435 oligo 40, ASV 721 oligo36, ASV 724 oligo43 and ASV 1309 oligo39) were unique in this sample. These results support recent findings indicating that UCYN‐A comprises a diverse group of ecotypes that may occupy different temporal niches in each oceanic region (Robicheau et al., 2023).

The diversity of the UCYN‐A community differed between the two consecutive years (Figure S4). In general, diversity was higher in most of the samples collected during La Niña than during El Niño, in the suboxic bottom of the shallow coastal station during La Niña. This was the only sequenced sample within the StSsW mass and had the lowest temperature (17.3°C) and oxygen (16.6 μM) and the highest nutrient concentrations (14.7 μM DIN; 3.3 μM PO_4_^−3^) of all the sequenced samples. This sublineage has shown its ability to proliferate in cold (10°C–20°C) and N‐rich waters (Cabello et al., 2020; Turk‐Kubo et al., 2021).

**FIGURE 5:**
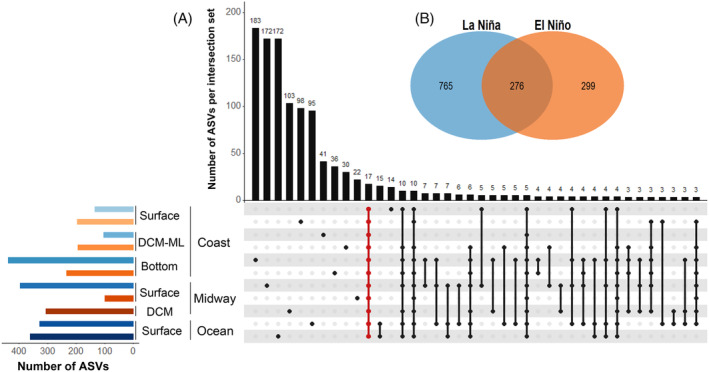
(A) UpSet plot: Distribution of ASVs across samples. The upper right bar chart indicates the number of ASVs shared by samples (the number of ASVs in each set appears above the bar), while the samples shared are indicated in the dot chart below the bar chart (the dots highlighted in red indicate ASVs shared by all samples). The lower left bar chart indicates the number of ASVs per sample; blue gradient, La Niña; orange gradient, El Niño. (B) Venn diagram: unique and shared ASVs between both ENSO phases.

Of 1340 ASVs, 17 ASVs representing 75% of the total sequences (16 ASVs assigned to oligo3 and 1 to oligo36) were common to all samples (Figure 5). This marked variability of the UCYN‐A community at the ASV level, with few shared ASVs along the transect and during both ENSO phases, suggests that a high number of ASVs are adapted to specific temporal niches in the ETNP off Mexico. For instance, only 20.6% of ASVs were shared between both ENSO phases: 1041 ASVs were recorded in six samples during La Niña, of which 57.1% were unique, whereas 575 ASVs were recorded in five samples during El Niño, of which 22.3% were unique. The highest number of unique ASVs was observed at the bottom of the shallow coastal station during La Niña (183 ASVs; 0.8% of the total sequences), followed by the surface of the midway and most oceanic stations (172 ASVs each; 0.9% of the total sequences), also during La Niña. The intense upwelling of nutrient‐rich waters during La Niña stimulated productivity in the euphotic zone, which could have promoted the proliferation of the UCYN‐A community.

**FIGURE 6:**
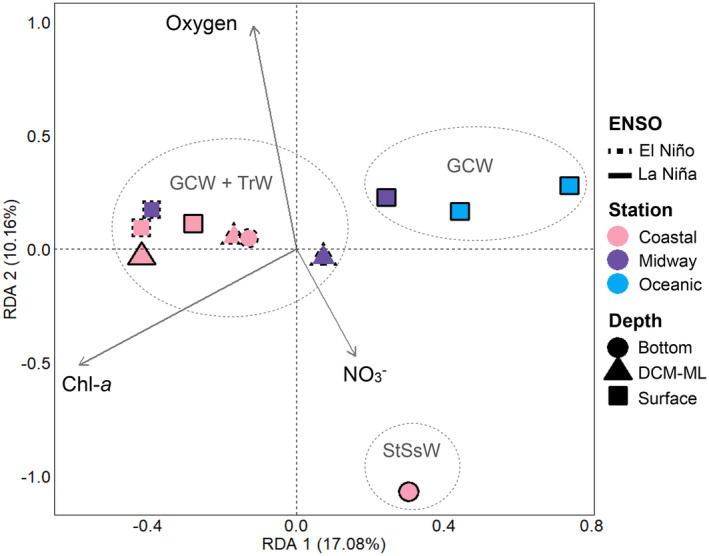
Redundancy analysis (RDA) plot of the samples (symbols) based on Hellinger‐transformed ASV abundances depicting the relationship between the UCYN‐A community and non‐collinear environmental variables (VIF < 10). Symbols: shape, depth; fill colour, sampling zone; border line, ENSO phase. Length and angle of arrows: extent of correlation between significant environmental factors (*p* < 0.1, 999 permutations) and RDA axes. Dotted lines surround samples within the same water mass. Chl‐*a*, chlorophyll‐*a*; NO_3_^−^, nitrate; DCM‐ML: deep chlorophyll‐*a* maximum or mixed layer; GCW, Gulf of California water; StSsW, Subsurface Subtropical water; TrW, Transitional water.

Three environmental variables (NO_3_^−^, chlorophyll‐*a* and oxygen) explained 35.7% of the UCYN‐A community variation and the first two RDA axes captured 27.2% of the variance of the dataset, suggesting that other factors might also be affecting the structure of this community in the study transect (Figure 6). The community from the surface samples of the midway and the two oceanic stations during La Niña, within the warm and well‐oxygenated GCW mass, was negatively correlated with the chlorophyll‐*a* concentration (*p* < 0.05). The community structure from most of the coastal samples of both ENSO phases and the surface sample of the midway station during El Niño, within the GCW and TrW masses, was positively correlated with high oxygen concentrations (*p* < 0.1) and negatively correlated with NO_3_^−^ (*p* < 0.05). In contrast, the community from the coastal bottom during La Niña, under the domain of the cold StSsW mass, was influenced by high NO_3_^−^ (7 μM) and low oxygen (16.6 μM) concentrations, indicating that UCYN‐A could tolerate these conditions (Mills et al., 2020; Turk‐Kubo et al., 2018).

### Presence of UCYN‐A in the Mexican Pacific ODZ

Diverse and active non‐cyanobacterial diazotrophs are already known in hypoxic and suboxic waters, such as the ETNP (Jayakumar et al., 2017), the ETSP (Fernandez et al., 2011), the Arabian Sea (Jayakumar et al., 2012) and the Baltic Sea (Farnelid et al., 2013), as well as the UCYN‐B clade in the ETSP off Peru (Loescher et al., 2014). In the present study, UCYN‐A was found for the first time at the suboxic bottom of a shallow coastal station (32 m depth) in the ETNP under the dominance of the cold, low‐oxygen and N‐rich StSsW mass. In addition, this bottom sample had the most diverse community and the highest number of unique ASVs of all sequenced samples in the transect studied (Figures S4 and S5), with 12 oligotypes belonging to UCYN‐A2 and 1 oligotype belonging to UCYN‐A3 (Figure 3).

Coastal upwelling systems and aphotic ODZs are potential habitats for non‐cyanobacterial diazotrophs (Moisander et al., 2017). Previous studies have reported N_2_ fixation rates in deep‐waters ODZs (Bonnet et al., 2013; Farnelid et al., 2013; Fernandez et al., 2011; Hamersley et al., 2011), supporting the hypothesis that low oxygen conditions and high surface productivity may favour N_2_ fixation by non‐cyanobacterial diazotrophs despite significant concentrations of DIN in these environments (Selden et al., 2021). The present punctual finding of UCYN‐A in a low‐oxygen coastal station in the Mexican Pacific during La Niña might suggest that the UCYN‐A/haptophyte symbiosis can also occupy shallow euphotic ODZs. Further evidence for the presence of UCYN‐A under these suboxic conditions is needed to support this assumption. On the other hand, this symbiosis cannot thrive in deeper aphotic ODZs, because the haptophyte needs light for photosynthesis.

Biological N fixation by UCYN‐A is not inhibited by the high availability of DIN (Turk‐Kubo et al., 2018). The UCYN‐A/haptophyte symbiosis has been found to rely on N_2_ fixation by UCYN‐A as a primary N source to meet much of the haptophyte's N needs in N‐replete waters, as the haptophyte does not assimilate NO_3_^−^ and only meets a small part of its N needs through NH_4_^+^ uptake (Mills et al., 2020). Therefore, it should not surprise that euphotic N‐rich shallower ODZs might allow the presence of UCYN‐A/haptophyte symbiosis, as occurred at this coastal station during La Niña.

Although this clade of cyanobacteria, in particular the UCYN‐A2 sublineage, is known to have a remarkable ability to proliferate under diverse oceanographic conditions as those present in this ODZ region, it is still unclear whether UCYN‐A can actively fix N in suboxic waters. More molecular data and measurements of biological N fixation rates are needed to assess the contribution of UCYN‐A to N cycling in ODZs.

## CONCLUSIONS

This study shows the presence of the UCYN‐A clade in the Mexican Pacific upwelling system under two different ENSO conditions. Although the absolute abundance of UCYN‐A was not affected by the ENSO phenomenon, its relative abundance and diversity were higher during La Niña than during El Niño along the study transect. ENSO forcing affects the distribution of water masses and, therefore, variables such as oxygen and nutrient availability, which regulate UCYN‐A community assemblage in this region.

UCYN‐A2 was by far the most abundant sublineage, with oligo3 being dominant along the study transect in both ENSO phases, whereas UCYN‐A1 (oligo1) and UCYN‐A3 (oligo39) were barely detected. These results suggest that the fluctuating oceanographic conditions induced by ENSO events do not affect the presence of oligo3 within UCYN‐A2 along the transect but do affect the occurrence of other less abundant oligotypes. In addition, compared to the other samples, a higher diversity of UCYN‐A was detected at the N‐rich suboxic bottom of the shallow coastal station; those were conditions promoted by La Niña, which induced an intense upwelling that brought the StSsW mass with oxygen‐deficient and nutrient‐rich cold waters towards the coast.

Although the distribution of UCYN‐A was associated with well‐oxygenated waters in this upwelling system, UCYN‐A was also detected in suboxic waters at the shallow coastal station. This is the first time that the presence of UCYN‐A has been reported in an ODZ. However, more extensive studies involving a larger number of samples under similar conditions are needed to know whether UCYN‐A can inhabit suboxic waters.

This study contributes to a deeper understanding of the distribution patterns of this clade of cyanobacteria under different climatic and oceanographic conditions. Further knowledge of the environmental and biogeochemical factors that affect UCYN‐A growth, distribution and activity, as well as the rates of biological N fixation by UCYN‐A in oxygen‐deficient marine systems may become increasingly pertinent given present concerns about deoxygenation in the world's oceans.

## AUTHOR CONTRIBUTIONS

**Cinthya Vieyra‐Mexicano:** Data curation (equal); formal analysis (equal); methodology (equal); writing – original draft (supporting); writing – review and editing (supporting). **Valeria Souza:** Resources (supporting). **Silvia Pajares:** Conceptualization (lead); data curation (equal); formal analysis (equal); funding acquisition (lead); investigation (lead); methodology (equal); project administration (lead); resources (lead); supervision (lead); validation (lead); writing – original draft (lead); writing – review and editing (lead).

## CONFLICT OF INTEREST STATEMENT

The authors declare no conflicts of interest.

## Supporting information


**Figure S1.** Vertical sections of oceanographic parameters during La Niña (left panels) and El Niño (right panels): (a) temperature (°C), (b) oxygen (μM), (c) chlorophyll‐*a* (mg/m^3^) and (d) salinity (g/kg^1^). Stations: C, coastal; M, midway 1 and 2; O, oceanic 1 and 2.
**Figure S2.** Vertical sections of nutrient concentrations (μM) during La Niña (left panels) and El Niño (right panels): (a) NH_4_^+^, (b) NO_2_^−^, (c) NO_3_^−^, (d) PO_4_^3−^. Stations: C, coastal; M, midway 1 and 2; O, oceanic 1 and 2.
**Figure S3.** Vertical profiles of the abundance of the UCYN‐A *nifH* gene from the sea surface to the bottom in the sampling stations during La Niña (blue lines) and El Niño (orange lines): (a) Coastal station; (b) Midway station, (c) Oceanic stations (O1: dashed line; O2: solid line). The circles indicate the sampling depths.
**Figure S4.** Alpha diversity indices of the UCYN‐A community: (a) Chao1, (b) Shannon, (c) Simpson 1‐D, (d) Faith's PD. Blue gradient, La Niña; orange gradient, El Niño; missing bars, samples not sequenced; DCM, chlorophyll‐a maximum; ML, mixed layer.
**Table S1.** ENSO phases, stations and sampling depths for molecular analyses, amplified samples, sequenced samples (marked with a dot), qPCR abundances of UCYN‐A *nifH* (mean ± standard deviation), water masses and environmental variables that were associated with the UCYN‐A community in the RDA analysis (oxygen ‐O_2_‐, Chlorophyll‐*a* ‐Chl‐*a*‐, nitrate ‐NO_3_^−^‐).

**Table S2.** Taxonomic assignment of ASVs obtained in the Mexican Pacific upwelling system by comparison with reference sequences of UCYN‐A oligotypes (Turk‐Kubo et al., 2017). NA: not assigned (see Excel file).

## Data Availability

Raw sequences are available at the NCBI Sequence Read Archive under the BioProject PRJNA882389. Scripts for sequence analysis are available as a Supporting file.
