# Supplementary material for: Distribution of the N2 ‐fixing cyanobacterium Candidatus Atelocyanobacterium thalassa in the Mexican Pacific upwelling system under two contrasting El Niño Southern Oscillation conditions
Source: Environ Microbiol Rep. 2024 Feb 13;16(1):e13237. doi: 10.1111/1758-2229.13237 (PMC10866059; doi:10.1111/1758-2229.13237)
Supplement: Supplementary file 1 — Figure S1. Vertical sections of oceanographic parameters during La Niña (left panels) and El Niño (right panels): (a) temperature (°C), (b) oxygen (μM), (c) chlorophyll‐a (mg/m3) and (d) salinity (g/kg1). Stations: C, coastal; M, midway 1 and 2; O, oceanic 1 and 2. Figure S2. Vertical sections of nutrient concentrations (μM) during La Niña (left panels) and El Niño (right panels): (a) NH4 +, (b) NO2 −, (c) NO3 −, (d) PO4 3−. Stations: C, coastal; M, midway 1 and 2; O, oceanic 1 and 2. Figure S3. Vertical profiles of the abundance of the UCYN‐A nifH gene from the sea surface to the bottom in the sampling stations during La Niña (blue lines) and El Niño (orange lines): (a) Coastal station; (b) Midway station, (c) Oceanic stations (O1: dashed line; O2: solid line). The circles indicate the sampling depths. Figure S4. Alpha diversity indices of the UCYN‐A community: (a) Chao1, (b) Shannon, (c) Simpson 1‐D, (d) Faith's PD. Blue gradient, La Niña; orange gradient, El Niño; missing bars, samples not sequenced; DCM, chlorophyll‐a maximum; ML, mixed layer. Table S1. ENSO phases, stations and sampling depths for molecular analyses, amplified samples, sequenced samples (marked with a dot), qPCR abundances of UCYN‐A nifH (mean ± standard deviation), water masses and environmental variables that were associated with the UCYN‐A community in the RDA analysis (oxygen ‐O2‐, Chlorophyll‐a ‐Chl‐a‐, nitrate ‐NO3 −‐). [file EMI4-16-e13237-s001.docx]

**Supplementary material**


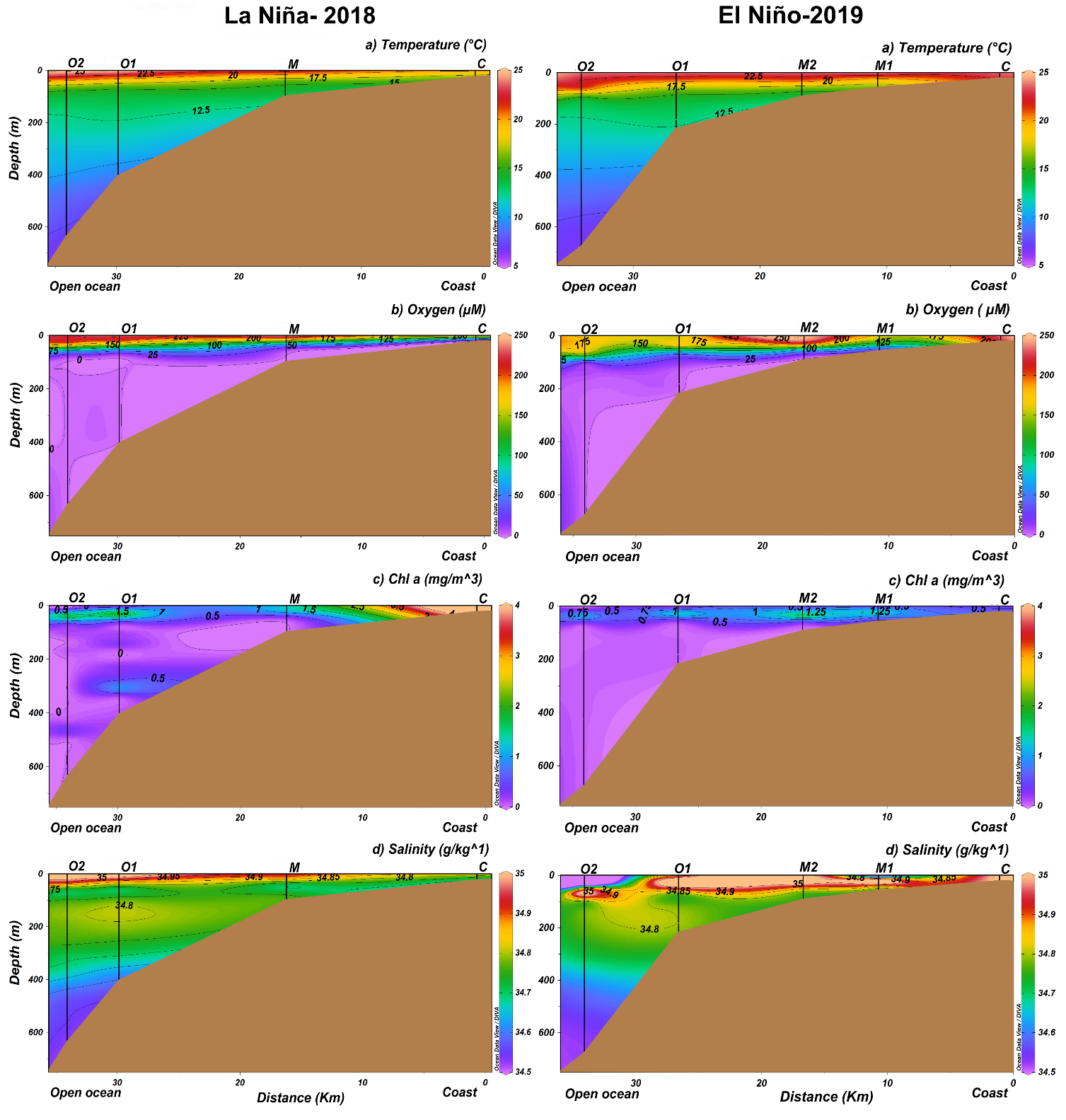


**Figure S1.** Vertical sections of oceanographic parameters during La Niña (left panels) and El Niño (right panels): a) temperature (ºC), b) oxygen (μM), c) chlorophyll-*a* (mg/m^3^), and d) salinity (g/kg^1^). Stations: C = coastal, M = midway 1 and 2, O = oceanic 1 and 2.


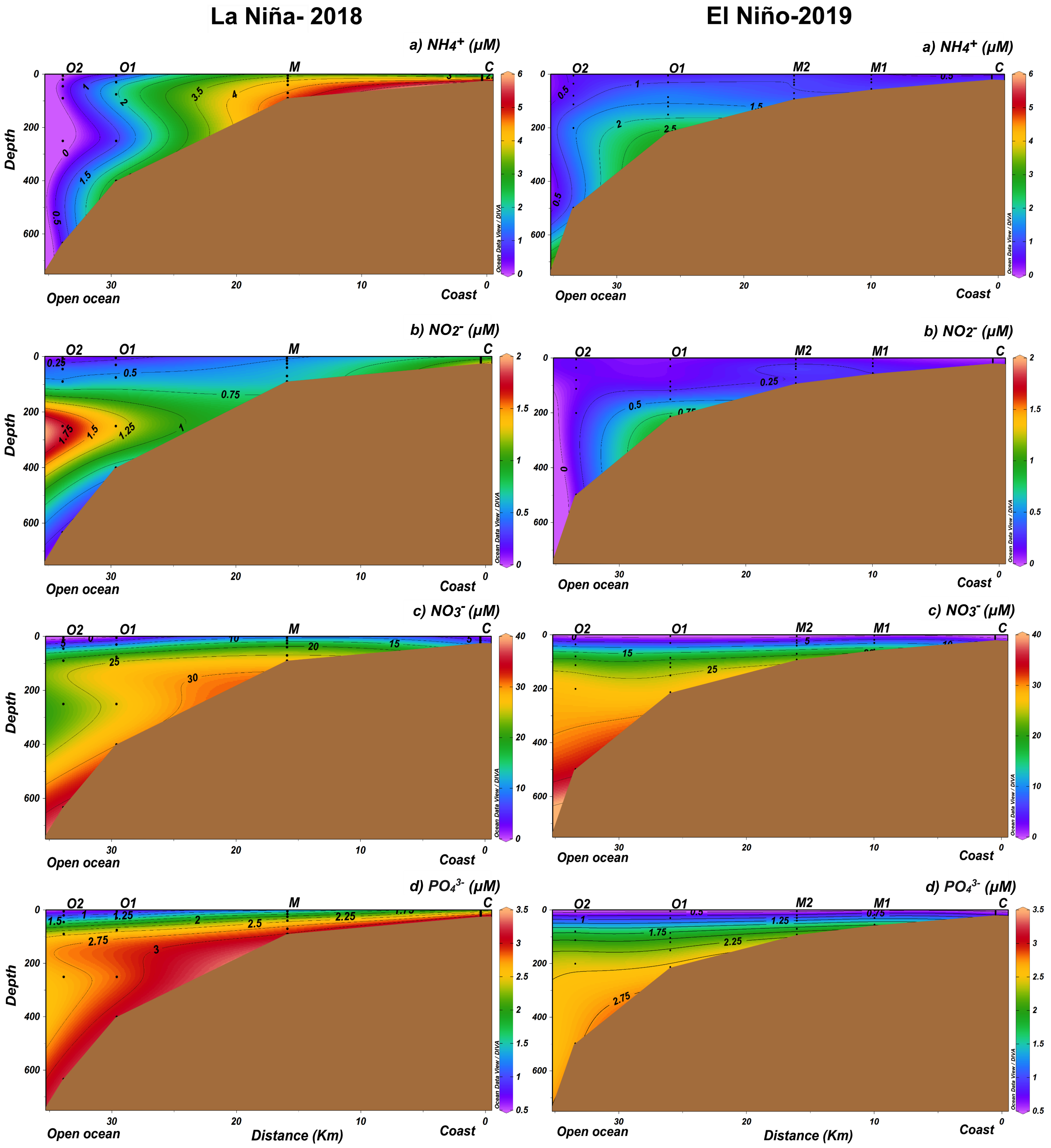


**Figure S2.** Vertical sections of nutrient concentrations (μM) during La Niña (left panels) and El Niño (right panels): a) NH_4_^+^, b) NO_2_^-^, c) NO_3_^-^, d) PO_4_^3-^. Stations: C = coastal, M = midway 1 and 2, O = oceanic 1 and 2.

**Figure S3.** Vertical profiles of the abundance of the UCYN-A *nifH* gene from the sea surface to the bottom in the sampling stations during La Niña (blue lines) and El Niño (orange lines): a) Coastal station; b) Midway station, c) Oceanic stations (O1: dashed line; O2: solid line). The circles indicate the sampling depths.

**Figure S4.** Alpha diversity indices of the UCYN-A community: a) Chao1, b) Shannon, c) Simpson 1-D, d) Faith´s PD. Blue gradient, La Niña; orange gradient, El Niño; missing bars, samples not sequenced; DCM, chlorophyll-a maximum; ML, mixed layer.

**Table S1.** ENSO phases, stations, and sampling depths for molecular analyses, amplified samples, sequenced samples (marked with a dot), qPCR abundances of UCYN-A *nifH* (mean ± standard deviation), water masses, and environmental variables that were associated with the UCYN-A community in the RDA analysis (oxygen –O_2_–, Chlorophyll-*a* –Chl-*a*–*,* nitrate –NO_3_^-^–).

| **ENSO** | **Station** | **Sampling depth (m)** | **Feature** | **Amplified samples** | **Sequenced samples** | **qPCR abundances** **UCYN-A *nifH***  **(copies mL^-1^)** | **Water mass** | **O_2_ (μM)** | **Ch-a**  **(mg m^-3^)** | **NO_3_^-^ (μM)** |
| --- | --- | --- | --- | --- | --- | --- | --- | --- | --- | --- |
| La Niña (2018) | Coastal (C) | 5 | Surface | Yes | ● | 306 ± 53.2 | TrW | 190 | 5.8 | 2.3 |
|  |  | 10 | DCM | Yes | ● | 233.7 ± 23.4 | TrW | 60 | 6.2 | 3.7 |
|  |  | 23 | Bottom | Yes | ● | 163.6 ± 22.3 | StSsW | 16.6 | 4.4 | 7 |
|  | Midway (M) | 5 | Surface | Yes | ● | 274.9 ± 48.7 | GCW | 226 | 1.3 | 5.6 |
|  |  | 22 | DCM | No |  | 198.8 ± 37.8 | TrW | 126 | 1.7 | 22.6 |
|  |  | 60 | BO | No |  | 0 ± 0 | StSsW | 5.2 | 0 | 23 |
|  |  | 93 | Bottom | No |  | 0 ± 0 | StSsW | 1.3 | 0 | 27 |
|  | Oceanic 1 (O1) | 5 | Surface | Yes | ● | 181.6 ± 31 | GCW | 214 | 0.27 | 0.3 |
|  |  | 30 | DCM | No |  | 98.9 ± 16.7 | TrW | 178 | 2.2 | 6.6 |
|  |  | 75 | BO | No |  | 0 ± 0 | StSsW | 4.1 | 0 | 25.5 |
|  |  | 250 | ODZ core | No |  | 0 ± 0 | StSsW | 0.44 | 0 | 24.2 |
|  |  | 400 | Bottom | No |  | 0 ± 0 | StSsW | 0.46 | 0 | 30.2 |
|  | Oceanic 2 (O2) | 5 | Surface | Yes | ● | 140.5 ± 23.2 | GCW | 211 | 0.04 | 0.2 |
|  |  | 45 | DCM | No |  | 80.1 ± 15.2 | TrW | 137 | 2 | 11.2 |
|  |  | 88 | BO | No |  | 0 ± 0 | StSsW | 4.3 | 0.1 | 24 |
|  |  | 250 | ODZ core | No |  | 0 ± 0 | StSsW | 0.4 | 0 | 21.2 |
|  |  | 650 | Bottom | No |  | 0 ± 0 | PIW | 0.3 | 0 | 36 |
| El Niño (2019) | Coastal (C) | 5 | Surface | Yes | ● | 176.8 ± 39.6 | GCW | 227 | 0.47 | 0.05 |
|  |  | 15 | Mixed layer | Yes | ● | 108.3 ± 18.5 | GCW | 226 | 0.51 | 0.1 |
|  |  | 23 | Bottom | Yes | ● | 119.3 ± 24.2 | GCW | 214 | 1.9 | 0.3 |
|  | Midway (M2) | 5 | Surface | Yes | ● | 217.7 ± 30 | GCW | 238 | 0.62 | 0.17 |
|  |  | 40 | DCM | Yes | ● | 88.5 ± 14.2 | TrW | 118 | 2.1 | 8.55 |
|  |  | 70 | BO | No |  | 0 ± 0 | StSsW | 5.9 | 0.1 | 22.5 |
|  |  | 93 | Bottom | No |  | 0 ± 0 | StSsW | 2.1 | 0 | 23 |
|  | Oceanic 2 (O2) | 5 | Surface | Yes |  | 114 ± 19.6 | GCW | 219 | 0.2 | 0.09 |
|  |  | 35 | DCM | No |  | 24 ± 7.7 | GCW | 192 | 1.7 | 3 |
|  |  | 125 | BO | No |  | 0 ± 0 | StSsW | 6 | 0 | 23.4 |
|  |  | 400 | ODZ core | No |  | 0 ± 0 | StSsW | 1.1 | 0 | 32.7 |
|  |  | 670 | Bottom | No |  | 0 ± 0 | PIW | 1 | 0 | 44.6 |

Abbreviations: DCM, deep chlorophyll-*a* maximum; BO, the base of the oxycline; ODZ, oxygen-deficient zone; GCW, Gulf of California water; PIW, Pacific Intermediate water; StSsW, Subtropical Subsurface water; TrW, Transitional water. The seafloor varied among stations (from 32 m depth in the coastal station C to 690 m depth in the most oceanic station O2).

**Table S2.** Taxonomic assignment of ASVs obtained in the Mexican Pacific upwelling system by comparison with reference sequences of UCYN-A oligotypes (Turk-Kubo et al., 2017). NA: not assigned **(see Excel file)**.
